# Reliability and Detectability of Emergency Management Systems in Smart Cities under Common Cause Failures

**DOI:** 10.3390/s24092955

**Published:** 2024-05-06

**Authors:** Thiago C. Jesus, Paulo Portugal, Daniel G. Costa, Francisco Vasques

**Affiliations:** 1 DTEC-UEFS, State University of Feira de Santana, Feira de Santana 44036-900, Brazil; tcjesus@uefs.br; 2 SYSTEC-ARISE, Faculty of Engineering, University of Porto, 4200-465 Porto, Portugal; danielgcosta@fe.up.pt; 3 INEGI, Faculty of Engineering, University of Porto, 4169-007 Porto, Portugal; vasques@fe.up.pt

**Keywords:** urban emergencies, urban planning, emergency management system, emergency detection, disaster recovery, emergency detectability

## Abstract

Urban areas are undergoing significant changes with the rise of smart cities, with technology transforming how cities develop through enhanced connectivity and data-driven services. However, these advancements also bring new challenges, especially in dealing with urban emergencies that can disrupt city life and infrastructure. The emergency management systems have become crucial elements for enabling cities to better handle urban emergencies, although ensuring the reliability and detectability of such system remains critical. This article introduces a new method to perform reliability and detectability assessments. By using Fault Tree Markov chain models, this article evaluates their performance under extreme conditions, providing valuable insights for designing and operating urban emergency systems. These analyses fill a gap in the existing research, offering a comprehensive understanding of emergency management systems functionality in complex urban settings.

## 1. Introduction

In the era of digital transformation, the concept of smart cities has emerged as a revolutionary approach to urban development. A smart city leverages information and communication technologies to enhance the quality of urban services, reduce costs, and improve the interaction between citizens and the government. These cities are characterised by a high degree of connectivity, automation, and data exchange, potentially leading to improved services efficiency and sustainability when pursuing better quality of life for their inhabitants [1].

However, the complexity and dynamics of smart cities also present unique challenges. One of the most critical issues is the impact of urban emergencies such as fires, floods, earthquakes, traffic accidents, and pandemics [2]. These emergencies can cause significant disruption to the city’s infrastructure and services, leading to economic losses and, in severe cases, loss of life [3]. Tackling urban emergencies has thus been promoted as a critical step in meeting the UN’s Sustainable Development Goals (SDG), particularly the SDG 11 [4,5,6].

In this context, an emergency management system (EMS) plays a vital role in a smart city’s resilience strategy, contributing to the safety and security of the city inhabitants. An EMS is expected to address a variety of urban emergencies, which will be detected and identified by a dedicated set of emergency detection units (EDUs). In short, an EDU is a multi-sensor device that will gather pertinent data according to the particular emergency scenario it was designed for. Moreover, a single EDU may contribute to the detection of multiple emergency types [7], with collected or processed data being disseminated through a mesh network until they reach a sink node. That node is responsible for the aggregation of the data, which will eventually consolidate this information with a base station or a cloud service. This basic operation flow has guided the development of EMS in recent years, with different levels of complexities and limitations [8,9,10,11].

Due to the large number of challenges around EMS when it comes to data acquisition, processing, and transmission, two important aspects have to be considered, although they are often neglected. First, EMS reliability comes as a probability that the system will function without failure for a specified period under given conditions [12,13,14]. In turn, detectability refers to the capability of an EMS to successfully detect a specific emergency. Therefore, when addressing both aspects, it is desired that an EMS will be able to operate continuously and reliably even in the face of unforeseen events, while also being capable of promptly and accurately identifying emergent threats. This combined perception of reliability and detectability forms the foundation of “emergency detection resilience”, which is a critical aspect when creating safer cities [15,16].

The reliability of an EMS, in addition to the individual reliability of its devices and the interaction between them, also depends on the reliability of the infrastructure in relation to the occurrence of an emergency. When an emergency occurs, it can have a profound impact on the infrastructure serving the EMS, potentially resulting in system failures. This vulnerability to failure can be exacerbated by the occurrence of common cause failures (CCFs), which significantly influence the reliability of an EMS. In general, CCFs manifest when multiple components of a system fail due to a shared underlying cause [17,18]. To illustrate, consider a scenario where an extreme weather event, such as a hurricane or flood, causes widespread power outages across a city [6,19,20]. In this case, the loss of electricity could lead to the simultaneous failure of multiple EMS components, such as communication networks, resulting in a collective system failure. Understanding and mitigating the risk of CCFs is therefore imperative in the design and operation of an EMS.

This article proposes the modelling of emergency management systems in smart city scenarios, encompassing the assessment of reliability and detectability, taking into account the effects of common cause failures in the system. In general, an EMS is expected to be composed of a wireless sensor network, where each node acts as an EDU that must collaboratively gather information from specific zones and deliver them to a sink node, that will handle the detection process. For that, we model the EMS network using a hierarchical paradigm composed of a Fault Tree and Markov chain models, which are analytically evaluated to determine the EMS reliability and detectability components in the form of metrics. This approach allows for the characterisation of generic EMSs, offering flexibility in terms of the quantity and types of emergencies that can be monitored simultaneously. Additionally, some experiments are conducted showing how to compute both metrics, assuming combinations of simulated emergencies. To the best of our knowledge, such an approach comprising the reliability and detectability of urban emergency management systems in an intertwined manner, and during extreme conditions, has not been proposed before.

In summary, the original contributions of this article can be succinctly delineated as follows:

- The conceptualisation and mathematical formulation of *emergency detectability*, leveraging *k*-coverage principles to exploit the dynamic nature of smart cities.
- The definition and mathematical formulation of the reliability of urban emergency management systems, based on the concept of emergency detectability. This includes a model of common cause failures and their systemic impacts.
- Refinement of dependability evaluation methodologies proposed in previous works, tailored to incorporate the newly proposed models of EMS reliability and emergency detectability. This approach provides a comprehensive, analytical, and quantitative assessment tool for generic EMSs, combining both reliability and detectability in a way not previously explored in the literature.
- Simulation results demonstrating the practical application of the detectability concept in assessing EMS performance in terms of reliability, particularly under extreme conditions.

The remainder of this article is structured as follows. Section 2 presents related works aimed at resilience of emergency management systems in smart cities. The proposed mathematical model to describe an EMS and its reliability assessment are presented in Section 3. Section 4 presents the conceptualisation of the detectability of an emergency. Experimental simulations are described in Section 5, followed by a comprehensive discussion about the achieved results and foreseen perspectives. Finally, conclusions are stated in Section 6.

## 2. Related Works

Emergency management systems in smart cities have gained considerable attention in recent years, given their pivotal role in ensuring the safety and resilience of urban communities. As cities have grown and become more complex environments, dealing with applications in smart cities scenarios has became also more complex. Several research works have been conducted, focused on investigating various aspects of EMS design, operation, and performance to address the evolving challenges posed by urban emergencies. This section provides an overview of the related works in this field, highlighting key studies and contributions.

Several studies have explored the conceptual framework and architectural design of EMSs in smart cities, aiming to develop comprehensive and effective systems for emergency detection and response. For example, a novel EMS architecture has been proposed, integrating heterogeneous sensor networks and addressing data fusion techniques to enhance situational awareness and decision-making in emergency scenarios [21,22,23]. Another important perspective of EMSs is their distributed implementation, leveraging advanced communication technologies, such as Internet of Things (IoT) and cloud computing, into the EMS infrastructure to improve responsiveness and scalability [16,24,25].

Another critical aspect of EMS research is the model and evaluation of system dependability under different operational conditions and emergency scenarios, particularly concerning reliability. Researchers have employed analytical models, simulation techniques, and empirical studies to assess system performance in real-world contexts [26,27,28]. Most works focus on describing strategies to improve EMS resilience, neglecting the necessity to properly assess the reliability level of the system, especially in critical situations, for which it is fundamental to accurately know the impact of hardware failures, communication disruptions, and environmental factors on system reliability, aiming to mitigate this impact [12].

Nowell et al. [29] discuss various strategies aimed at achieving resilience in EMSs, many of which are centred around redundancy but extend beyond mere reliance on backup resources. These strategies encompass cross-functionality, involving the utilisation of a unit that serves multiple roles or functions within the system. Additionally, duplication is presented as a type of redundancy characterised by having multiple ways for performing the same task and/or multiple individuals or technologies assigned to the same function. Another highlighted strategy is cross-check, which entails procedural verification, information verification, and tactical verification. While addressing crucial aspects of EMS reliability, this work does not provide methods to assess the impact of these measures on the EMS.

Abdel-Basset et al. [30] propose an integrated framework to evaluate the performance of smart disaster response systems through some group of criteria that are measured, such as Smart servicing, Smart update, Appropriateness, Authenticity, Versatility. However the EMS reliability is not, indeed, computed. The referred criteria are weighted using the analytic hierarchy process (AHP) and ranked by their relevance degree to that EMS, through Technique in Order of Preference by Similarity to Ideal Solution (TOPSIS) and Vlse Kriterijumska Optimizacija Kompromisno Resenje (VIKOR).

In [26], EMS resilience is measured by a hybrid model integrating System-Theoretic Accident Model and Processes (STAMP) with a dynamic Bayesian network (DBN) to manage emergency failure risks and enhance the system’s resilience in the process of emergency operations. This is an interesting solution since it is feasible for the failure analysis of complex emergency systems. However, the authors do not consider the effect of common cause failures or the emergencies detectability.

Another interesting solution for EMS assessment is presented [12]. In this case, the authors propose a Petri Net-based approach for the modelling and analysis of a healthcare system in IoT infrastructures. The proposed models allow assessing measures, such as system availability, cost, and recovery time objective. However, while this work offers valuable insights, it does not address the effects of common cause failures or the detectability of emergencies, besides limiting its applicability to specific scenarios.

The identification and mitigation of common cause failures (CCFs) are crucial tasks in EMSs, as these failures simultaneously impact multiple components or subsystems, resulting in systemic failures [18,31]. CCFs pose a significant threat to system reliability, particularly in safety-critical systems like emergency management systems [32,33]. Numerous studies have explored CCF modelling techniques and risk assessment methodologies to enhance system robustness [18,34,35]. However, there remains a gap in the literature regarding the specific challenges posed by emergency management systems in smart cities, given their safety-critical nature and geographical complexities [1,36,37].

In summary, the presented related works have made valuable contributions to the field of reliability assessment of emergency management systems for smart cities. However, there are notable gaps. The existing literature lacks a comprehensive assessment of system reliability in critical situations, particularly those exacerbated by common cause failures. Moreover, the detectability of emergencies, a crucial aspect of EMS, has not been adequately addressed. Furthermore, the applicability of some proposed solutions is limited to specific scenarios, thereby restricting their utility in the diverse and complex environments characteristic of smart cities.

Our approach presents a substantial advancement over existing methods for evaluating the reliability of emergency management systems in smart cities. While previous works have focused on improving EMS resilience through various strategies, many lack a structured method for quantitatively assessing the system reliability and emergencies detectability, particularly when facing common cause failures. Furthermore, some existing methodologies provide scenario-specific assessment of reliability, failing to offer a holistic or comprehensive analysis across different emergency scenarios.

The proposed approach leverages previous evaluation methodologies that integrate Fault Tree and Markov chain models to analytically evaluate systems. Our contribution relies on properly adapting these approaches to encompass the evaluation of reliability of generic EMSs, considering the effects of common cause failures on the system. This provides a robust framework for quantitatively assessing EMS performance across a wide range of emergency scenarios, offering broader applicability and more nuanced insights into EMS operations. Unlike other methods, our approach is based on the novel concept of emergencies detectability, a critical aspect of EMSs often overlooked in existing research.

By combining both reliability and detectability assessments, our method offers a complete and integrated evaluation of EMS performance. This allows for the precise identification of potential weaknesses within the system and offers actionable insights for system improvement. Additionally, our approach’s adaptability makes it suitable for various smart city environments and emergency scenarios, providing a versatile tool to help guide the development of more resilient and effective emergency management systems, ultimately enhancing the safety and resilience of smart cities.

## 3. Fundamentals and Proposed Model

Let us consider that a smart city is served by an EMS that monitors a set of distinct urban emergencies, encompassing fires, floods, earthquakes, traffic accidents, pandemics, and more. The detection of each emergency relies on a set of emergency detection units, with each unit potentially contributing to the detection of multiple emergencies. The EMS operates as a distributed system, utilising a wireless sensor network, where sensor nodes serve as EDUs strategically positioned throughout the cityscape.

The task of monitoring entire cities presents inherent complexity, as it is impractical to infer the overall behaviour of a region solely from measurements taken at specific sites. For instance, observing rainfall in certain regions does not necessarily imply that it is raining citywide. This highlights the challenge of extrapolating measurements across vast areas. Consequently, our detection model relies on subdividing the city into smaller zones called zones of interest (ZI), which may encompass neighbourhoods or parishes, for more detailed analysis and monitoring.

Thus, the total region *R* of a city can be defined as a set of *r* zones of interest. So, let $E=\left\{e_{1},e_{2},\ldots ,e_{m}\right\}$ be the set of emergencies that an EMS can monitor. For each zone of interest $z_{i}\in R$, where i=1,⋯,r, it is assigned a subset of emergencies $E_{i}=\left\{e_{1},\cdots ,e_{h}\right\}$ ($E_{i}\subseteq E$). This subset includes a selection of emergencies $e_{j}$ (j=1,⋯,h) that the system is equipped to detect and address within that specific zone $z_{i}$. Notably, each zone of interest may present distinct sets of emergencies, influenced by geographical, social, and historical factors unique to that particular area.

This geographical arrangement implies that each emergency will affect the EDUs differently. In the event of an emergency, damage may be inflicted on the city’s physical structure, including the infrastructure of the EMS. For instance, detection units may cease to function after prolonged exposure to a fire, or they may be swept away to remote locations in the event of a hurricane. These effects may be defined by the city geography. For example, EDUs positioned in high-relief zones are likely not to be damaged in the event of floods. Similarly, the occurrence of a fire in one neighbourhood will not influence the EDUs deployed in another, more distant neighbourhood. This means that each emergency is associated with the reliability of a subset of EDUs, which can have a greater impact on the reliability of the EMS. Hence, we say that each EDU has a susceptible set of emergencies, representing those capable of causing damage to the EDU upon their occurrence. An EDU may be susceptible to none, some, or all emergencies within its operational scope.

Therefore, notice that emergencies of the same type, but in different zones, are interpreted as different emergencies. That is, if a fire occurs in zone $z_{1}$ and another in zone $z_{2}$, although they may have the same configuration parameters, they should be treated as two distinct emergencies; after all, the geographical characteristics differ from one zone to another, so the means and mechanisms to detect and mitigate emergencies in different zones should also be different.

**Figure 1:**
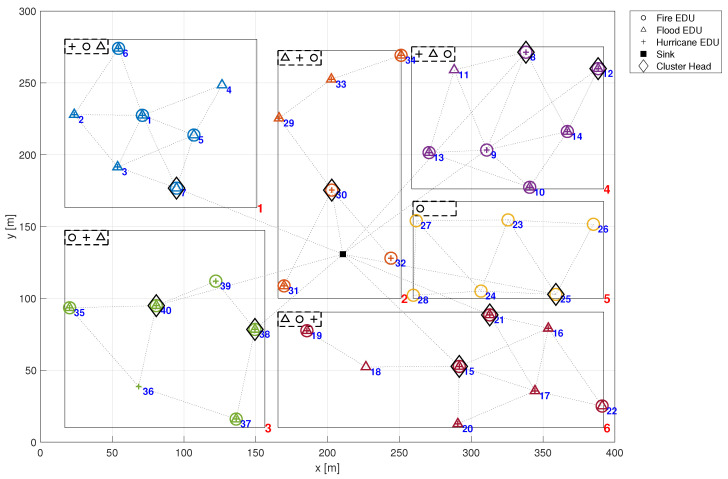
Deployed scenario.

Figure 1 provides a visual representation of a deployed emergency management system within a specified region, depicted as a 400 m × 300 m rectangle, further partitioned into six distinct zones. In this figure, both the *x*-axis and *y*-axis represent distance, measured in meters (m). Both the city region and the individual zones are represented by rectangular models. This approach does not compromise the method’s precision since it allows the definition of non-rectangular city or zone boundaries through the union of multiple rectangular regions. This allows to model emergency management systems in a variety of urban settings. By approximating complex, non-uniform city shapes as a union of disjoint rectangular sub-regions, the proposed model can be applied to any urban environment, regardless of its layout. In Figure 1, each zone of interest is uniquely identified by a red numerical label located in the bottom right corner of its corresponding rectangle, denoting its zone number. Moreover, each zone is tasked with monitoring at least one emergency type, including fire, flood, and hurricane, in this case.

The symbols within each zone in Figure 1 denote the specific types of emergencies monitored by the respective EDUs (identified by blue numerical labels). For example, EDU 1 located in zone 1 is equipped to detect all emergency types: fire (represented by a circle), flood (depicted as a triangle), and hurricane (illustrated by a plus sign). It is noteworthy that each ZI presents a rectangular dashed box in its the top left corner, delineating the emergencies detectable within that zone. Further details regarding the detectability of emergencies are discussed in the following section.

## 4. Emergency Detectability

In this article, we introduce the concept of emergency detectability, which can be illustrated by the following scenario. Suppose that an application may have a single EDU capable of collecting data from a specific zone, with a reliability of 90%. However, the low variety of sources makes the detection unrepresentative. It is reckless to try to describe the behaviour of a large area with just one EDU. This detection is not informed enough.

On the other hand, if a zone has 10 EDUs, but with a reliability of 10%, this means that the application will not be active for long, and therefore, the detection system is also not reliable to perform this service. Thus, the user must indicate the minimum number of sources and know the minimum expected reliability of that specific emergency.

Therefore, the EMS detectability can be defined as the application ability to monitor an emergency using the minimum necessary data sources while ensuring the system reliability. This emergency monitoring must be performed within a specific zone. This concept is elucidated as a particular case of *k*-coverage, which refers to a measure of redundancy indicating the level of coverage provided by sensor nodes in a given area [38,39]. In essence, *k*-coverage denotes the minimum number of sensors required to guarantee that a target area is covered or monitored adequately [40,41].

Similarly, we define that an emergency is detectable within a given zone if there exists a required minimum number of EDUs actively monitoring that emergency within the zone, with the capability to transmit their monitoring data to the sink node. Put simply, an emergency is considered detectable within an EMS if the system possesses a sufficient number of operational hardware components capable of sensing the emergency, and an available network infrastructure to relay the sensed data to a central processing centre.

Both reliability and detectability are defined as percentage measures of probabilities. Reliability R(t) can be defined as the probability that the system performs its tasks without failure until time *t* [14]. Detectability D(t) is defined in this article as weighted reliability, proportional to the number of emergencies the system can detect at time *t*.

In order to formally define detectability, let us establish the relaxed notation u∈e indicating that the EDU u∈U monitors the emergency *e*. Similarly, if the EDU u∈U is located within a zone *z*, it is denoted as u∈z. Thus, we can define the coverage function from Equation (1), indicating that a set of EDUs U⊂U can cover a zone *z* regarding the emergency *e*, if there are at least *k* EDUs in *U*, located in zone *z*, monitoring the emergency *e*. Equation (2) indicates the existence of a re-transmission path in the network allowing an EDU to deliver its messages to the sink. Finally, Equation (3) indicates that an emergency is detectable if there are coverage and communication conditions provided by the EMS:

(1) $$coverage\left(k,e,z,U\right)=\left\{\begin{array}{cc} 1, & \exists U_{z}^{e}\subset U\;\;\;|\;\;\;\forall u\in U_{z}^{e},u\in z,u\in e,\left|U_{z}^{e}\right|\geq k\\ 0, & \mathrm{otherwise} \end{array}\right.$$

(2) $$path\left(u,sink\right)=\left\{\begin{array}{cc} 1, & \mathrm{if}\;\mathrm{exists}\;\mathrm{network}\;\mathrm{path}\;\mathrm{connection}\;\mathrm{from}\;u\;\mathrm{to}\;\sin k\;\mathrm{node}\\ 0, & \mathrm{otherwise} \end{array}\right.$$

(3) detectable(k,e,z,U)=coverage(k,e,z,U).path(u,sink)

Understanding the detectable condition of an emergency, the EMS detectability D(t) is formulated as the EMS reliability R(t) weighted by the proportion of detectable emergencies within the system. This formulation is depicted in Equation (4):

(4) $$D\left(t\right)=R\left(t\right)\cdot \frac{\sum_{e\in E}^{\;}[detectable(k,e,z,U)]}{\left|U\right|}$$

A high level of emergency detectability implies that the EMS can reliably identify and respond to emergencies promptly and accurately, thereby facilitating timely intervention and mitigating potential risks to public safety and infrastructure. Conversely, low emergency detectability indicates inefficiencies or limitations in the system’s ability to detect and respond to emergencies effectively, which can lead to delays in emergency response efforts and increased vulnerability to adverse outcomes.

Modelling and evaluating emergency detectability is of paramount importance, especially in complex urban environments and dynamic emergency scenarios. This metric estimates how an EMS will perform even under various failure events. This requires careful management of factors such as the number and reliability of detection units deployed in the monitoring area, the coverage and granularity of monitoring, and the resilience of communication and information-sharing mechanisms within the EMS network. These considerations are addressed directly or indirectly in Equations (1)–(4). It becomes evident that the effectiveness of an EMS crucially depends on not only on its ability to detect emergencies but also on its reliability in doing so. The concept of emergency detectability, as elucidated, emphasises the importance of having an optimal balance between the number of data sources and their reliability to ensure efficient emergency monitoring within a designated zone. Thus, the proposed EMS reliability evaluation methodology.

### Reliability Assessment of Emergency Management System

The proposed reliability assessment methodology in this article addresses a significant gap in the literature concerning emergency management systems (EMSs) designed for smart cities operating under common cause failures. Building upon previous methodologies [27,42], we refined our approach by introducing a novel process for generating Network Failure Conditions (NFCs) tailored to the specific requirements of EMSs in smart cities. Additionally, we incorporated common cause failure modelling into the Fault Tree analysis, resulting in a more comprehensive and practical methodology that offers a complete mathematical solution for such scenarios. Our approach considers detectability failures, which occur when there is a breakdown in communication to the sink node from a minimum number of EDUs located within a zone in the cityscape, all monitoring the same emergency. Such failures may arise from ordinary hardware malfunctions or common cause failures.

**Figure 2:**
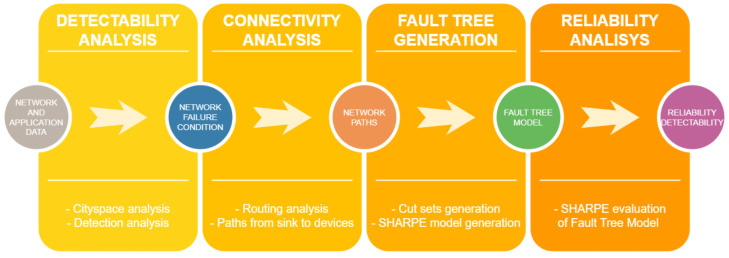
Steps of the proposed evaluation methodology.

The flowchart of the proposed evaluation approach is illustrated in Figure 2, showing the method in four distinct stages: the innovative *detectability analysis*, *connectivity analysis*, the novel *Fault Tree generation* incorporating common cause failures, and *reliability analysis*. Examining the proposed methodology in reverse, i.e., from the final stage back to the first, the *reliability analysis* is carried out using the SHARPE (Symbolic Hierarchical Automated Reliability and Performance Evaluator) tool [43], which calculates a hierarchical model of a Fault Tree embedded with Markov chains. It is worth noting that this method is automated, meaning that once the initial data are provided, no additional user intervention is needed for the dependability assessment.

The hierarchical model is constructed in the previous stage, the *Fault Tree generation*. This methodology assumes a network with a tree structure rooted at the sink node, which handles the emergency management application by receiving data from the EDUs. Therefore, the EMS will fail if the information related to a monitored emergency cannot be delivered to the sink. This failure can result from issues with the EDUs forming the *Network Failure Condition* (NFC), a logical expression specifying which nodes or combinations of nodes must fail to cause an application failure. The nodes involved in the NFC are known as *essential nodes*.

**Figure 3:**
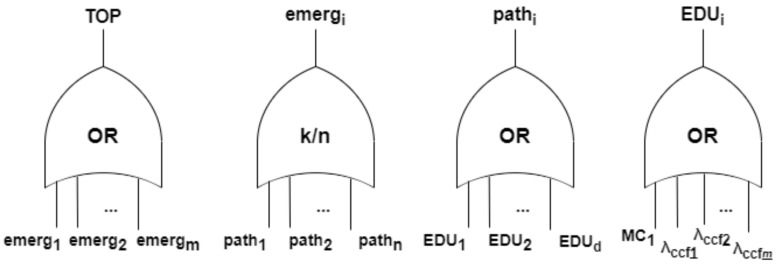
Fault Tree models of EMS (TOP), emergencies, paths, and EDUs.

The NFC is used to construct a Fault Tree, and thus it is equivalent to the EMS failure. In this context, an EMS failure occurs when the NFC evaluates to *true*, which matches the TOP event of the Fault Tree also evaluating to *true*. Figure 3 illustrates the Fault Tree structure, which is incorporated in the methodology.

**Figure 4:**
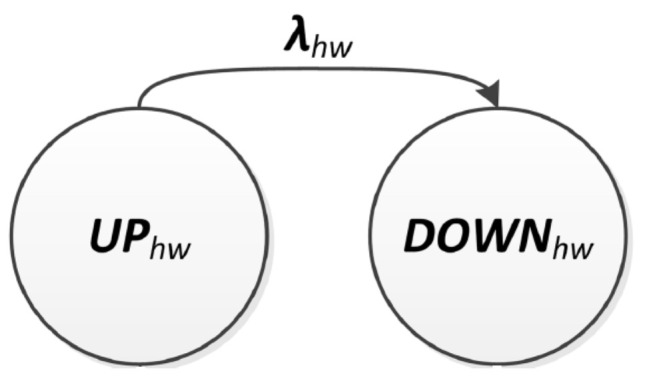
Hardware Markov chain model.

A single emergency fails, i.e., becomes undetectable, if a minimum number of paths connecting the sink to each node monitoring that emergency fail. A path fails if any EDU in the path from the node to the sink fails. Ultimately, an EDU fails if its hardware (modelled by a Markov Chain) fails or if an emergency triggers a common cause failure associated with that EDU. As mentioned, hardware failures must be assigned to the reliability function, derived from evaluating the Markov chain as depicted in Figure 4. The Markov chain describes the component behaviour through two states: UP (operational) and DOWN (failed). The transition between these two states is defined by the failure rate ($\lambda_{hw}$).

In the *connectivity analysis* stage (the second phase in Figure 2), the paths from NFC nodes to the sink are represented in the Fault Tree based on the routing strategy. This requires an understanding of the NFC of the network under consideration.

Finally, the *detectability analysis* is performed by verifying, for each emergency in each zone, how many and which EDUs must be functional once a single emergency is detectable. For this, *k*-out-of-*n* gates (voting gates, k−oo−n or k/n gates) are used, indicating that a failure occurs if at least *k* among the total of *n* elements fail. If all emergencies monitored by the EMS are detectable, then the EMS is considered to be functional. If any emergency fail, then the EMS fails.

In the presented evaluation methodology, besides the emergency detectability, we propose and model another concept: the conditional detectability. Considering that an EMS can manage more than one emergency simultaneously, the occurrence of a given emergency $e_{i}$ in a zone may disable the monitoring of some kind of emergencies in that zone. However, the EDUs in other zones may still be active and reachable, making it possible to manage other emergencies $e_{j}$ (j≠i). In this way, the reliability of the application can be calculated conditional on the occurrence of a given emergency. In other words, if an emergency $e_{i}$ occurs, we are interested in knowing what other emergencies can still be detected and what is the reliability of this new application.

The experimental results presented in the next sections shed light on this discussion and provide a deeper understanding of the proposed approach.

## 5. Experimental Results and Discussion

This section presents the results of a series of simulations conducted to evaluate the reliability and detectability of an emergency management system in smart cities under common cause failures. The simulations were developed in the MathWorks MATLAB platform, while the reliability assessment was performed using the SHARPE tool. Therefore, simulation scenarios were set up in a virtual environment designed to replicate real-world smart cities as accurately as possible, taking into account the complex nature of urban environments and the diverse range of emergencies that can occur. Thus, a variety of emergency detection units were placed throughout various zones of interest within the cityscape, each responsible for the detection and management of a specific set of emergency situations.

The efficacy of the application was evaluated based on a hierarchical routing strategy. This approach allows to leverage the inherent territorial divisions of a city into zones (neighbourhoods). Within each zone, EDUs might communicate via a mesh network, ensuring seamless information is delivered to designated EDUs serving as Cluster Heads (CHs). These CHs maintain direct connections with the network’s sink node (probably remotely located), forming a common scenario encountered in real-world applications [44,45].

For local communication within a zone, short-range technologies such as Wi-Fi, ZigBee, 6LoWPAN, WirelessHART, MiWi, and SNAP can be utilised. These options offer benefits in terms of cost-effectiveness and are well suited for nodes communication in close proximity within individual zones. Conversely, long-range technologies with high data rates like 5G/LTE and Weightless-W are better to be employed to transmit large volumes of data from the CH to a sink node located remotely. This segmentation of communication technologies optimises efficiency and resource utilisation [44,46,47,48].

The simulated scenarios are assessed to present results focused on several key aspects: the calculation of emergency detectability, the overall reliability of the application, and the conditional reliability given the occurrence of specific combinations of emergencies. These results provide valuable insights into the effectiveness of the EMS in managing emergencies in smart cities, particularly in situations where common cause failures occur. The following subsections detail the experimental setup, the results obtained, and the analysis of these results.

### 5.1. Overall EMS Reliability

**Figure 5:**
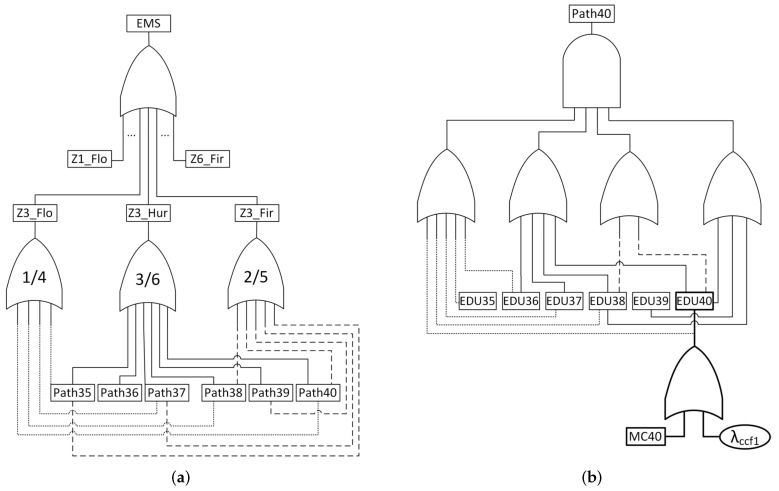
Overall Fault Tree model of the EMS in Figure 1.

The first presented result is to show how to assess the overall application reliability of an emergency management system. For this, let us consider the city already described in Figure 1. In that case, the city has six zones, each one with a few EDUs, some of which are acting as cluster heads. The reliability of an EMS executing over this infrastructure depends on the ability of a minimum amount of EDUs associated with each emergency to deliver their information to the sink node. Following the methodology described in Section 4, the EMS failure network condition is implemented in a Fault Tree integrated with Markov Chain reliability models of individual nodes. Figure 5 shows part of that Fault Tree. This model is used to assess the EMS reliability with and without the effect of the common cause failures ($\lambda_{ccf}$). In order to compute the dependability without the effect of CCF, these failure rates must also be set to zero.

Computing the reliability of the entire emergency management system entails assessing the probability of the network failure condition occurring across the entire application. In this context, the NFC is defined by the presence of at least $k_{i}$ active and reachable EDUs (those situated along a path to the sink node) in each designated zone $z_{i}$. While the required number of EDUs for emergency detection may differ from one zone to another and is potentially configurable, for uniformity and ease of explanation, we will consider this number to be constant across all zones. Therefore, a minimum of four active and reachable EDUs per zone are necessary to ensure the detectability of a specific emergency.

In Figure 5a, the failure conditions within zone 3 are illustrated. This zone is equipped with EDUs capable of monitoring fire, flood, and hurricanes. Specifically, there are six EDUs designated for hurricane monitoring (EDUs 35,36,37,38,39,40). Given that the predetermined minimum number of active EDUs required to detect an emergency is four, it follows that for a hurricane to go undetected, the sink node must cease receiving information from at least three out of these six EDUs in zone 3. Similarly, for a fire emergency to become undetectable in zone 3, the failure of paths associated with at least two out of five EDUs (35,37,38,39,40) is required. Likewise, for flood detection, the failure of paths associated with at least one out of four EDUs (35,37,38,40) is necessary. These communication breakdowns leading to the undetectability of an emergency can occur either due to the failure of an EDU itself or due to failures in intermediate nodes along the path to the sink. An EDU failure may result from hardware malfunctions, such as a broken electronic component, which is modelled in this article using a Markov Chain as shown in Figure 4. Additionally, common cause failures affecting a group of EDUs can contribute to their failure. It is important to note that similar reasoning must be applied to the other zones (1,2,4,5,6) to describe the failure scenarios within the entire system.

An illustration of path modelling is provided in Figure 5b, depicting potential failure scenarios where information from EDU 40 fails to reach the sink. Four distinct paths are outlined: {40−35−36−37−38−sink}, {40−36−37−38−sink}, {40−39−38−sink}, and {40−38−sink}. The failure of all these paths is necessary to prevent EDU 40 from communicating with the sink. It is important to note that the sink node, assumed to be immune to failure, is not depicted in the Fault Tree. Additionally, in Figure 5b, the failure modelling of an EDU, specifically EDU 40, is shown. Such failures may occur due to a Markov Chain-modelled hardware failure (MC40) or common cause failures. One such CCF, denoted as $\lambda_{ccf1}$, is represented in the diagram.

**Figure 6:**
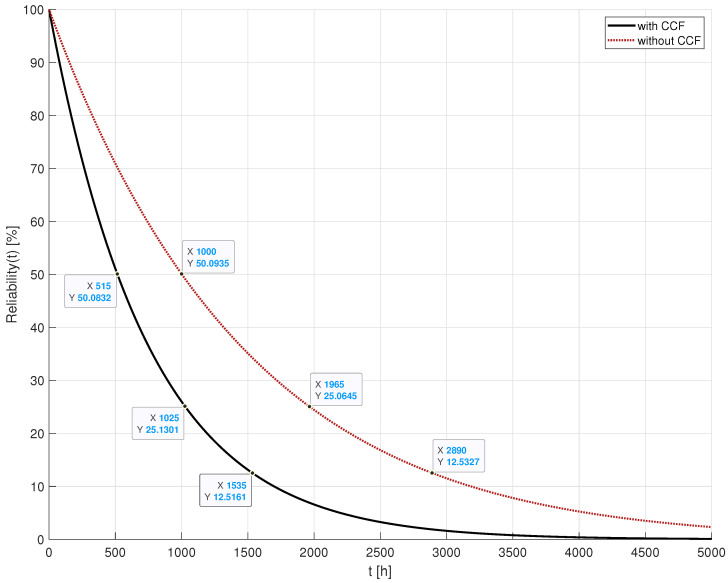
Reliability assessment.

The model under consideration was assessed by examining failure rates, denoted as $\lambda_{hw}$ for hardware failures, $\lambda_{ccf1}$ for common cause failures due to fires, $\lambda_{ccf2}$ for CCFs due to floods, and $\lambda_{ccf3}$ for CCFs due to hurricanes. The respective rates are $\lambda_{hw}=2.2831\times 10^{-5}/h$, $\lambda_{ccf1}=3.4722\times 10^{-4}/h$, $\lambda_{ccf2}=2.3148\times 10^{-4}/h$, $\lambda_{ccf3}=7.7160\times 10^{-5}/h$. To provide context, these values suggest an expected occurrence of one hardware failure every five years, a fire every four months, a flood every six months, and a hurricane every 18 months. Additionally, the radio communication range was set at 75 m. These parameter configurations are based on the experimental parameter settings discussion in [27] and the empirical timing of emergency occurrences. All these parameters remain constant across all simulation scenarios. The evaluation of these scenarios in terms of system reliability was conducted using the SHARPE tool [43]. The outcomes of this evaluation are depicted in Figure 6, which highlights the impact of CCFs on the emergency management system reliability. The data tips on the graph in this figure display the values for the *x*-axis and *y*-axis, which represent the time (*t*), measured in hours, and the reliability value (R(t)), expressed as a percentage, respectively. The comparison in Figure 6 contrasts a scenario unaffected by common cause failures with one influenced by them. As expected, the scenario without CCFs exhibited superior reliability. This is attributed to the nature of CCFs, which simultaneously impair multiple devices, making them non-functional. The impact of CCFs is notably significant as evidenced by the system reliability diminishing approximately twice as rapidly when subjected to these failures as can be verified in Figure 6.

It is worth noting that, for the sake of simplicity, we assumed identical values for common cause failure rates across all zones, without loss of generality. However, in reality, each zone within a city possesses unique characteristics and may present varying rates for the same type of emergency. For example, zones situated near rivers or the sea are more prone to flooding compared to higher ground areas. This variability can be effectively accommodated within the methodology proposed in this article, as it sets the CCF rates based on groups of EDUs rather than by the type of emergency. By doing so, the approach accounts for the diverse risk profiles inherent to different geographical locations within a city.

Building upon the understanding of a city’s diverse nature, the subsequent simulation focuses on evaluating reliability in response to conditional emergency occurrences. This analysis aims to assess the dynamic detectability of the EMS.

### 5.2. EMS Reliability under Emergency Occurrences

The simulations presented herein were designed to assess the reliability of the emergency response within the context of different emergency scenarios. They aim to analyse the dynamic capacity of the EMS to effectively detect such events, even under conditions that might be considered hazardous or catastrophic. The emphasis of this evaluation is placed on the ability of the EMS to promptly identify emergencies as they unfold, thereby illustrating the application and utility of the concept of emergency detectability.

**Figure 7:**
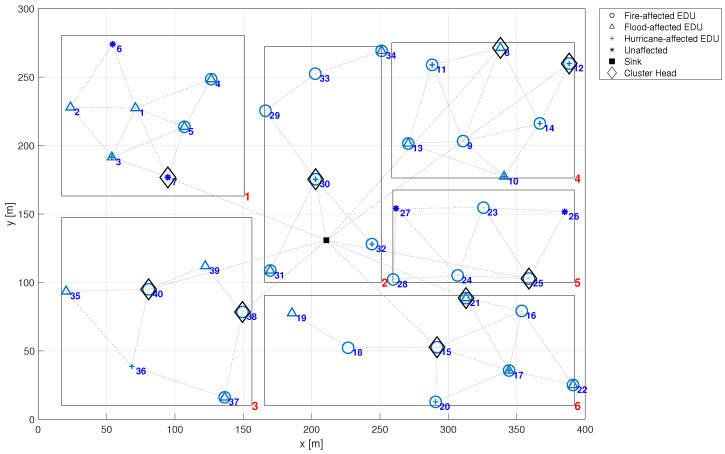
EDUs susceptibility to the different emergencies.

Hence, it is considered that certain known emergencies manifest at specific time intervals. Over time, EDUs susceptible to that emergencies will fail due to a common cause failure, altering the configuration of detectable emergencies at that time. Such occurrences potentially impact the system reliability and consequently, its detectability. In this example, we consider the EMS depicted in Figure 1, with its EDUs susceptible to various emergencies as delineated in Figure 7. In this figure, both the *x*-axis and *y*-axis represent distance, measured in meters (m). The symbols presented in the latter figure denote the type of emergency that leads each EDU to failure. For instance, EDU 33 in zone 2 is only susceptible to the Fire emergency. Conversely, in zone 3, EDU 35 is only susceptible to Flood emergency, while EDU 36 is only susceptible to Hurricane emergency. Notice that certain EDUs may be affected by multiple types of emergencies, exemplified by EDU 17 in zone 6, which is affected by Fire, Flood and Hurricane. On the other hand, some EDUs remain unaffected by any emergency, such as EDU 27 in zone 5.

**Figure 8:**
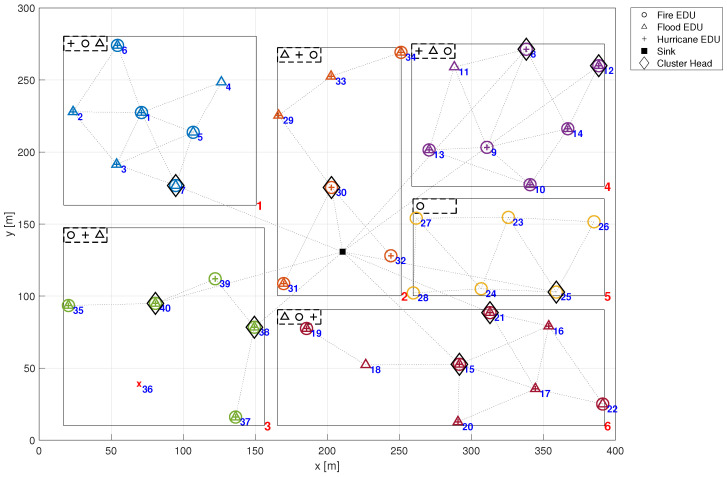
Scenario at t=250 h.

Let us consider a situation where a hurricane commences in zone 3 at time t=250 h. Referring to Figure 7, it becomes evident that only EDU 36 is impacted by this emergency, making it non-operational. This state can be seen in Figure 8, where an indication of a red ‘*x*’ is displayed at the position of EDU 36. However, it is noteworthy that the remaining EDUs in zone 3 retain their ability to detect fire, flood, and hurricanes as evidenced by the symbols in the rectangle at the top left corner of the zone as illustrated in Figure 8.

**Figure 9:**
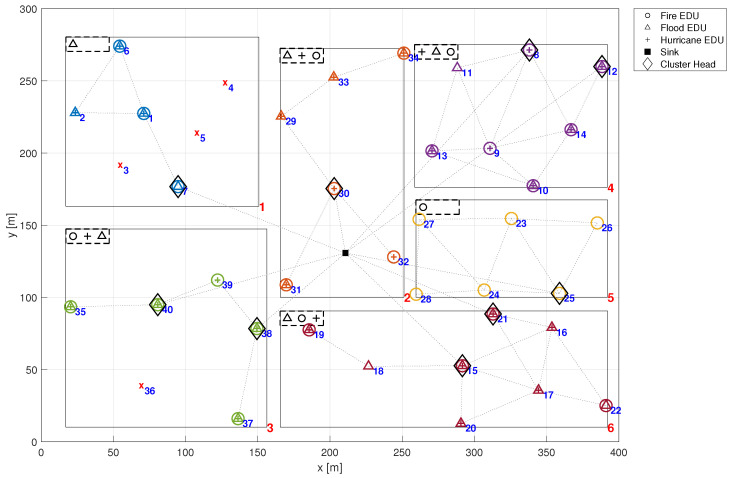
Scenario at t=500 h.

**Figure 10:**
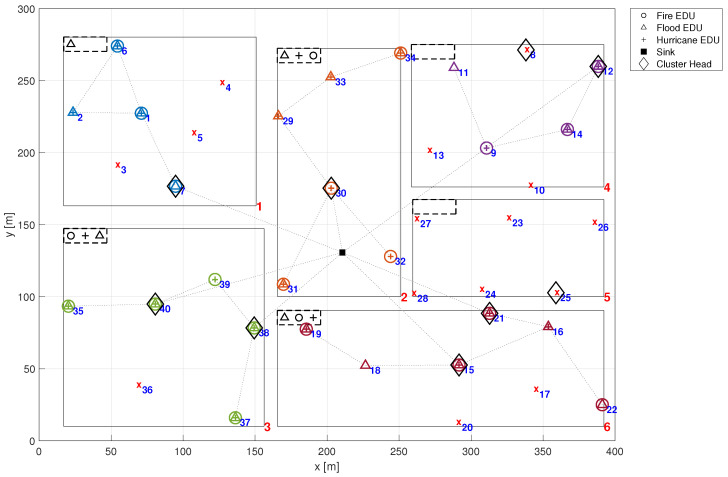
Scenario at t=1000 h.

Now, suppose that the hurricane moved and achieved zone 1 at time t=500 h, also causing a fire. In this case, besides EDU 36 which failed at t=250 h, EDUs 3, 4 and 5 also fail. Consequently, Fire and Hurricane can no longer be detected in zone 3, as shown in Figure 9. Moving forward, at time t=1000 h, let us assume that the hurricane reaches zones 5 and 6, resulting in a flood in zone 4 and fire in zone 5 as collateral damage (see Figure 10). This scenario characterises a catastrophic situation, where the proper functioning of the EMS is crucial. In this scenario, EDUs 8, 10 and 13 in zone 4 fail, leading to the loss of all emergency detection capabilities in this zone. Similarly, in zone 5, all EDUs fail (23, 24, 25, 26, and 27). Meanwhile, in zone 6, EDUs 17 and 20 also fail. However, this does not affect zone detectability. It is worth noting that the timeline of these events may appear to be taking too long. This decision regarding the configuration of the simulation scenario was deliberate, without loss of generality, aimed at enhancing the subsequent data visualisation of the graphical reliability analysis by distributing the occurrences more broadly.

**Figure 11:**
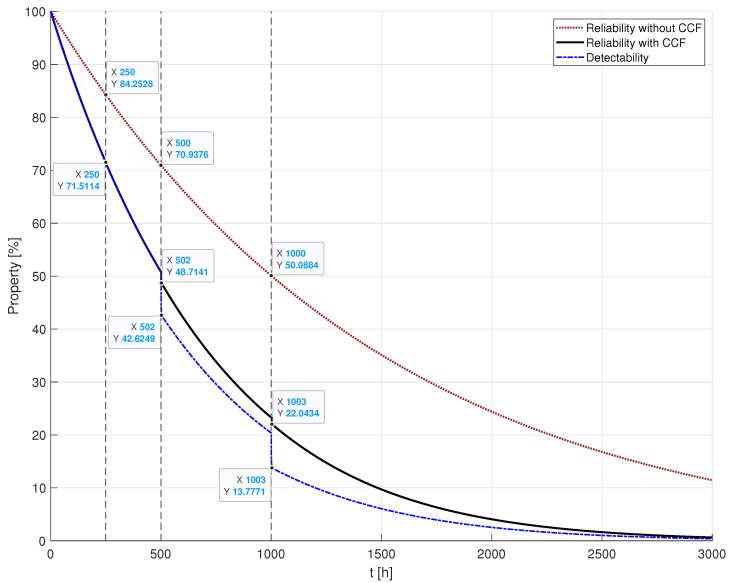
Reliability assessment.

The reliability analysis of the described scenario is depicted in Figure 11. The graph shows the time (*t*) on the *x*-axis, measured in hours, and the reliability value (R(t)) on the *y*-axis, expressed as a percentage. This figure presents the EMS reliability in contexts without and with common cause failures, alongside the EMS detectability. The reliability values of the system without CCF remain consistent with those presented in Figure 6, where no failures are induced, serving as a baseline for comparison. Conversely, the reliability values of the EMS affected by CCF reflect the outcome of system analysis within the evolving catastrophic scenario described. Meanwhile, EMS detectability—a metric introduced in Section 4—assesses the system’s capacity to identify multiple emergencies, even when some have already taken place.

Upon examining Figure 11, several insights emerge. Notably, the degrading impact of CCFs on system reliability is evident when juxtaposed with the CCF-free response of the EMS. Around t=1000 h, a simple subtraction calculation reveals that the reliability of the EMS under the CCF effect (22.0434%) experiences a decline of nearly 30% compared to its reliability without CCF (50.0884%), underscoring a critical need for enhancements during the EMS design phase to optimise system deployment. Additionally, it is notable that the system reliability remains unchanged at t=250 h. Despite the occurrence of a Hurricane emergency at that time, its effect is restricted to a single EDU, thereby not compromising EMS detectability.

However, at t=500 h, the EMS loses the ability to detect two emergencies in zone 1, resulting in a detectability decrease of approximately 6% compared to reliability. Finally, the catastrophic scenario at t=1000 h exhibits a similar loss of reliability compared to the scenario at t=500 h, although with a more pronounced detectability decrease of approximately 8%. This is attributed to the high number of undetected emergencies at t=1000 h, even with the presence of numerous operational EDUs. For example, in zone 4, despite these operational EDUs monitoring all emergencies, their collective efforts prove insufficient for detection. While this has a minimal impact on reliability, it significantly impacts detectability.

The analysis presented in this section underscores the criticality of reliability and detectability assessments in an EMS. The results demonstrate the detrimental effects of common cause failures, which significantly compromise both the robustness and the responsiveness of EMSs. Such insights are useful for city authorities and emergency response agents, as they provide a clearer understanding of urban dynamics during emergencies. This knowledge is pivotal in guiding the design and implementation of EMS to ensure they are not only resilient but also agile in their response to unforeseen events. Consequently, our findings suggest the necessity of a strategic approach to EMS development, one that prioritises continuous improvement and adaptability, thereby enhancing the overall safety and security of urban populations.

## 6. Conclusions

In conclusion, this article has addressed the critical issues surrounding the reliability and detectability of emergency management systems in smart cities. By introducing the concept of emergency detectability and its relationship with *k*-coverage, we have provided a framework for understanding and evaluating the effectiveness of emergency monitoring systems. We highlight the importance of optimising the deployment of sensor nodes and ensuring reliability to enhance the detectability of emergencies within specific zones. Through this conceptual framework, we have laid the groundwork for future research aimed at developing more resilient and efficient EMSs in smart cities.

Moreover, our analysis underscores the significance of considering common cause failures and their impact on system reliability. By acknowledging the vulnerabilities posed by CCFs, we can better design and implement EMS architectures that are robust and resilient in the face of unforeseen challenges. Additionally, our discussion emphasises the need for comprehensive reliability assessment methodologies that account for the complexities of urban environments and the dynamic nature of emergency scenarios. That way, this article contributes to the ongoing efforts to develop smarter, more responsive emergency management systems that safeguard urban communities and enhance overall resilience in the face of adversity.

As future research related to this article, digital twins could be designed and developed for simulating real-world EMS applications, providing valuable insights into system performance and reliability under various scenarios. Furthermore, the development of models to estimate the latency between emergency occurrence, detection, and system response time could enhance our understanding of EMS effectiveness and operational capabilities during emergencies. 

## Data Availability

No new data were created or analyzed in this study. Data sharing is not applicable to this article.
